# The Electric Brush in the Treatment of Locomotor Ataxia

**Published:** 1882-07

**Authors:** 


### THE ELECTRIC BRUSH IN THE TREATMENT OF LOCOMOTOR ATAXIA.

In the first number of the *Neurologisches Centralblatt*, issued in January, we find an important memoir by Dr. Rumpf, of Düsseldorf, on the treatment of locomotor ataxia with the electric brush. It is impossible to deny the relevancy of the statements if true. It is impossible to overestimate the importance of the fact alleged if confirmed. It is asserted by Dr. Rumpf that this almost incurable disease is arrested by applying the electric brush along the back and limbs. Our readers, of course, know that the electric brush is a bundle of fine wires, through which the faradic current is applied to the skin. That the faradic current applied to a part affects the circulation in distant parts, has been shown by Rumpf in another memoir. He has ascertained that a mild faradic current, applied on one side, causes a dilatation of the vessels on the other side. A fact like this throws light on the asserted power of the electric brush over posterior spinal sclerosis. But to the facts: The electric brush, applied to the back and legs of an ataxic of ten years' duration for the space of two months, produced an immediate amelioration, and such restoration of power and control that the patient resumed work. One year after his cure, Dr. Rumpf exhibited the patient to the Düsseldorf Medical Society, when the only remaining symptom was absence of the tendon reflex of the knee. He has a number of cases similarly improved, but awaits the development of sufficient time to determine the reality of the cures.

So simple a procedure as brushing over the back and limbs with an electric brush may at once be resorted to by large numbers of our readers. We hope that the new expedient will be given a proper trial and the results announced.—*Medical Times.*

